# Detection of Bourbon Virus-Specific Serum Neutralizing Antibodies in Human Serum in Missouri, USA

**DOI:** 10.1128/msphere.00164-22

**Published:** 2022-05-24

**Authors:** Gayan Bamunuarachchi, Houda Harastani, Paul W. Rothlauf, Ya-nan Dai, Ali Ellebedy, Daved Fremont, Sean P. J. Whelan, David Wang, Adrianus C. M. Boon

**Affiliations:** a Department of Medicine, Washington University School of Medicine in St. Louis, St. Louis, Missouri, USA; b Department of Molecular Microbiology, Washington University School of Medicine in St. Louis, St. Louis, Missouri, USA; c Department of Pathology and Immunology, Washington University School of Medicine in St. Louis, St. Louis, Missouri, USA; d Department of Biochemistry and Molecular Biophysics, Washington University School of Medicine in St. Louis, St. Louis, Missouri, USA; e Program in Virology, Harvard Medical School, Boston, Massachusetts, USA; University of Michigan—Ann Arbor

**Keywords:** Bourbon virus, seroprevalence, human serum, glycoprotein, monoclonal antibody, virus neutralization

## Abstract

Bourbon virus (BRBV) was first discovered in 2014 in a fatal human case. Since then it has been detected in the tick Amblyomma americanum in the states of Missouri and Kansas in the United States. Despite the high prevalence of BRBV in ticks in these states, very few human cases have been reported, and the true infection burden of BRBV in the community is unknown. Here, we developed two virus neutralization assays, a vesicular stomatitis virus (VSV)-BRBV pseudotyped rapid assay and a BRBV focus reduction neutralization assay, to assess the seroprevalence of BRBV neutralizing antibodies in human sera collected in 2020 in St. Louis, MO. Of 440 human serum samples tested, three (0.7%) were able to potently neutralize both VSV-BRBV and wild-type BRBV. These findings suggest that human infections with BRBV are more common than previously recognized.

**IMPORTANCE** Since the discovery of the Bourbon virus (BRBV) in 2014, a total of five human cases have been identified, including two fatal cases. BRBV is thought to be transmitted by the lone star tick, which is prevalent in the eastern, southeastern, and midwestern United States. BRBV has been detected in ticks in Missouri and Kansas, and serological evidence suggests that it is also present in North Carolina. However, the true infection burden of BRBV in humans is not known. In the present study, we developed two virus neutralization assays to assess the seroprevalence of BRBV-specific antibodies in human sera collected in 2020 in St. Louis, MO. We found that a small subset of individuals are seropositive for neutralizing antibodies against BRBV. Our data suggest that BRBV infection in humans is more common than previously thought.

## INTRODUCTION

Emerging and reemerging infectious diseases cause substantial global health and socioeconomic burdens and have a significant impact on human and animal life (1). Viral pathogens, like Ebola virus, severe acute respiratory syndrome coronavirus-2 (SARS-CoV-2), influenza virus, and Chikungunya virus, are among the most prominent emerging zoonotic infections (2). Many zoonotic diseases are transmitted by arthropod vectors such as mosquitos and ticks and are an important cause of human morbidity and mortality in the United States. Ticks are the main vector that transmit pathogens in the United States, including Powassan virus, Colorado tick fever virus, Heartland virus (HRTV), and Bourbon virus (BRBV).

BRBV belongs to the family *Orthomyxoviridae*, genus *Thogotovirus.* BRBV is a negative-sense segmented RNA virus whose genome is composed of 6 gene segments (3). Segment 4 encodes the viral envelope glycoprotein (GP) that is necessary for virus attachment and entry into cells (4), and it is the main target for virus-neutralizing antibodies (5). A total of five human cases of BRBV infection, including two fatal cases, have been reported since the discovery of this virus in 2014. The first case of BRBV infection was an adult male patient from Bourbon County, KS, USA. The patient was hospitalized with febrile illness and later died from renal failure and acute respiratory distress syndrome. Subsequent culturing and next-generation sequencing of the blood of this patient identified BRBV (6). In 2017, a state park official from Missouri was diagnosed positive for BRBV. This patient later died of respiratory failure and cardiac complications (3, 7). Both patients had reported tick exposure and bites prior to becoming ill (3, 6).

BRBV is isolated from lone star ticks (Amblyomma americanum) (8–11). These ticks are abundant and aggressive human-biting ticks that are widely distributed across the eastern, southeastern, and midwestern United States (9, 12). Rabbits fed on by BRBV-infected ticks developed high titers of antibody to the virus, suggesting that the lone star tick is a competent vector of BRBV (11). BRBV-neutralizing antibodies have been identified in different wild animal species. Using a plaque reduction neutralization test (PRNT), 50% and 86% seroprevalence of BRBV-neutralizing antibodies was found in sera from raccoons and white-tailed deer in Missouri, respectively (13). Moreover, 56% seroprevalence was observed in white-tailed deer sera from North Carolina (14). These observations demonstrate that the rate of infection of wild animals is significant, raising questions as to the true infection rate and clinical burden of BRBV in humans.

Serology can provide an important benchmark on population immunity against pathogens. However, to date, there have been no serosurveillance studies assessing the seroprevalence of BRBV-specific neutralizing antibodies in humans in the United States or the world. Thus, the objectives of our study were to develop BRBV neutralization assays and measure the human seroprevalence of BRBV infection. We found that nearly 1% of the human sera, obtained in 2020 in St. Louis, MO, contained BRBV-neutralizing antibodies. These data suggest that BRBV infection in people is more common than previously thought.

## RESULTS

### Construction of a replication-competent VSV expressing BRBV GP.

**FIG 1:**
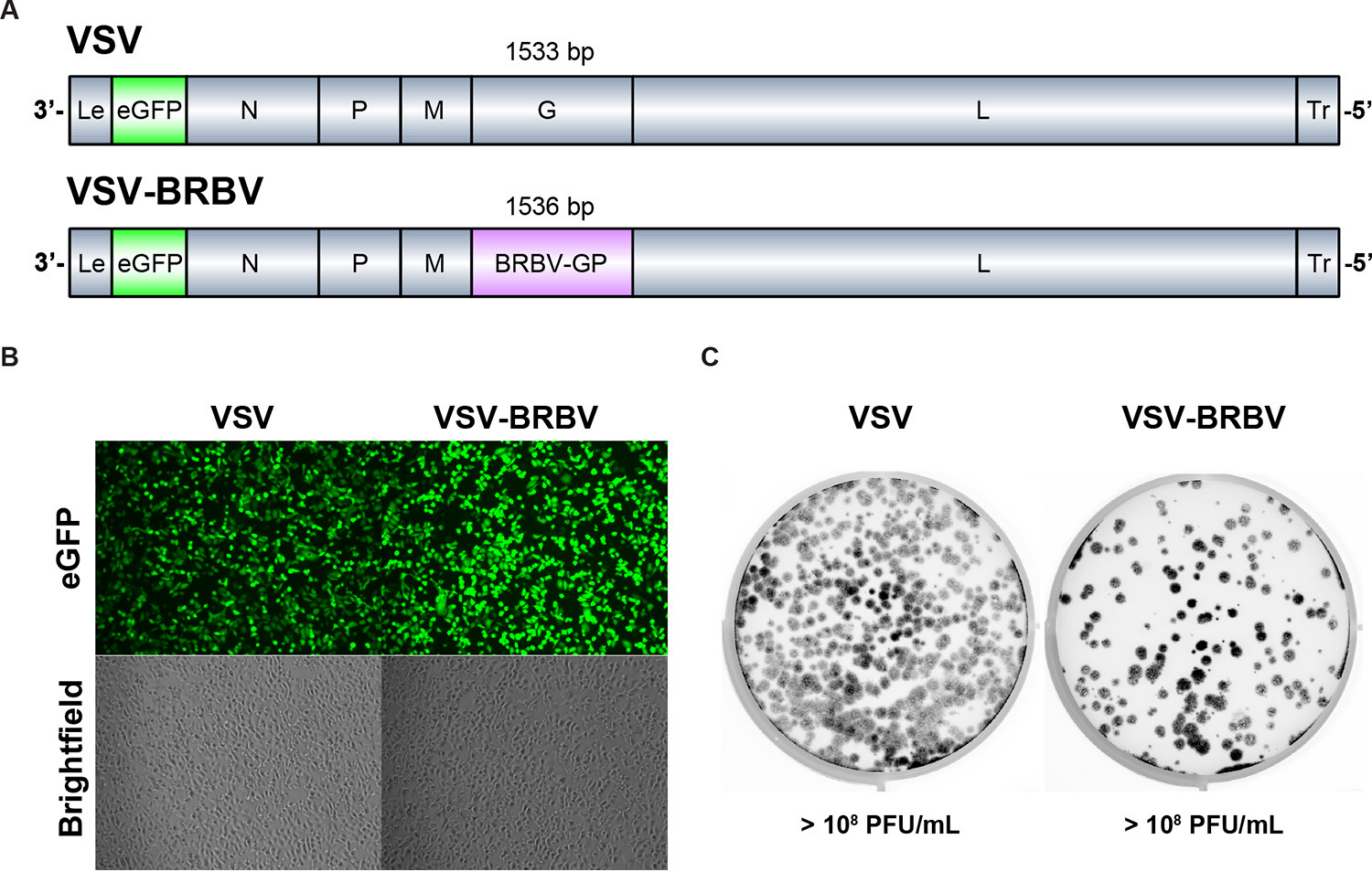
Generation of a replication-competent VSV expressing eGFP and BRBV GP. (A) Genome schematic. Genes in the viruses are to scale (except the leader [Le] and trailer [Tr]). N, nucleoprotein; P, phosphoprotein; M, matrix protein; G or GP, glycoprotein; L, large protein or polymerase. Glycoprotein gene length excludes the stop codon. (B) Vero-CCL81 cells were infected with VSV or VSV-BRBV at an MOI of 1, and images were acquired 6 hpi. (C) Representative plaque assay of VSV or VSV-BRBV on Vero-CCL81 cells. Images were acquired 24 hpi.

Since authentic BRBV is a biosafety level 3 (BSL3) pathogen, we sought to develop a tool to study aspects of the BRBV life cycle at reduced biocontainment. To do this, we replaced the glycoprotein gene in a molecular clone of enhanced green fluorescent protein (eGFP)-expressing vesicular stomatitis virus (VSV) with the GP gene of BRBV (VSV-eGFP-BRBV; referred to here as VSV-BRBV) (Fig. 1A). VSV-BRBV was capable of robust eGFP expression in Vero-CCL81 cells and grew to high titers (>10^8^ focus-forming units [FFU]/mL) with plaque size similar to that of VSV containing its native glycoprotein (Fig. 1B and C).

### Development of humanized monoclonal antibodies against GP of BRBV.

**FIG 2:**
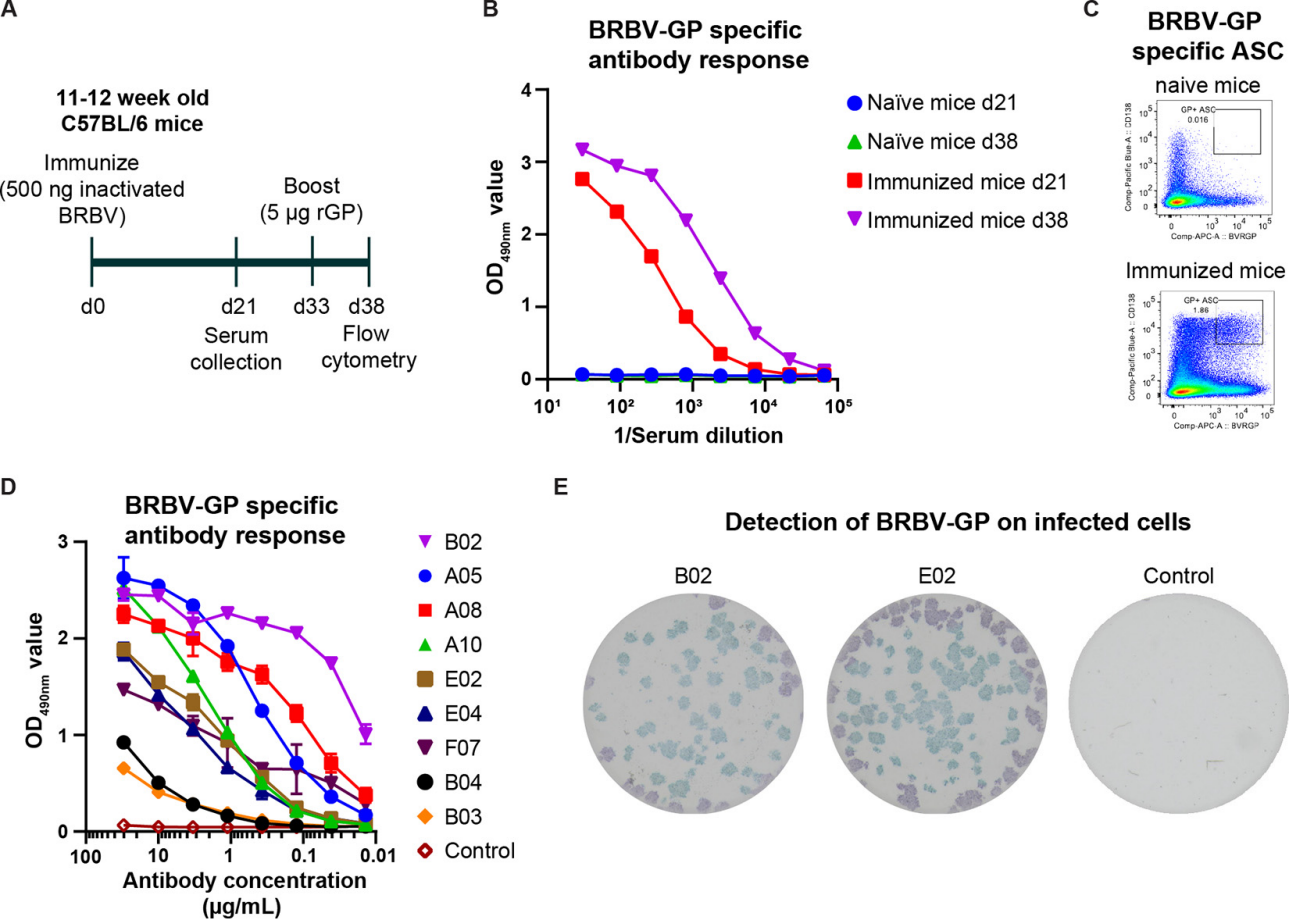
Generation and characterization of humanized monoclonal antibodies against BRBV GP. (A) Immunization regimen. Eleven- to twelve-week-old C57BL/6 mice were immunized intramuscularly with 500 ng BPL-inactivated BRBV, followed by boosters with 5 μg of rGP of BRBV 33 days later. Sera were collected at days 21 and 38. Spleens were harvested 5 days after the booster (day 38) (B) IgG serum Ab ELISA for rGP of BRBV for naive and immunized mice at days 21 and 38. Results are averages from two independent experiments. (C) Representative plots of BRBV GP ASC (CD4^−^ CD19^+^ IgD^lo^ CD95^+^ GL7^−^ CD138^+^ BRBV GP^+^ live singlet lymphocytes) in naive mice (top) and BRBV-immunized mice (bottom). (D) ELISA measuring binding of nine MAbs to rGP of BRBV. Each line represents a different antibody, and the results are averages from two independent experiments. (E) Binding of MAb B02 and E02 to BRBV-infected cells. Vero cells were infected with ~100 infectious units of BRBV for 24 h and fixed with 5% formalin, and virus-infected cells were visualized by incubating the cells with 2 to 3 μg/mL of MAbs B02 and E02 or a control. Images are representative of 2 experiments.

Eleven- to twelve-week-old C57BL/6 mice were immunized intramuscularly with inactivated BRBV followed by a single intramuscular booster with recombinant BRBV GP (rGP) 33 days later. Sera were collected 21 and 5 days after the first and second immunizations, respectively (Fig. 2A) and used to measure GP-specific antibody responses by enzyme-linked immunosorbent assay (ELISA). Compared to mice after the primary immunization, mice given boosters of rGP of BRBV showed a ~6-fold increase in serum antibody titers for binding to BRBV rGP (Fig. 2B). Five days after the booster, the spleens from one immunized animal and a control animal were isolated and processed for detection and isolation of BRBV GP-specific B cells. Nearly 2% of the antibody-secreting B cells (ASC) (CD4^−^ CD19^+^ IgD^lo^ CD95^+^ GL7^−^ CD138^+^) were specific for BRBV GP (Fig. 2C). No BRBV GP-specific ASC were detected in the naive control animal. The BRBV GP-labeled B cells were then single cell sorted by fluorescence-activated cell sorting (FACS) into 96-well plates containing lysis buffer. Amplification of variable heavy (VH) and variable light kappa and lambda (Vκ and Vλ, respectively) genes from a single B-cell-sorted 96-well plate showed ~70% (66/94) productive V domains, which were later grouped into nine different clonal groups based the identity of their V and J alleles and the CDR3 region. One representative VH and VL gene from each clonal group were cloned into human IgG1 expression vectors, transfected into HEK293 cells, and purified. These monoclonal antibodies (MAbs) are referred to as BRBV-GP-A05, -A08, -A10, -B02, -B03, -B04, -E02, -E04, and -F07. Purified MAbs were tested for specificity and affinity to rGP by ELISA (Fig. 2D). All nine antibodies were specific for BRBV GP, and three (BRBV-GP-A05, -A08, and -B02) demonstrated binding affinity to BRBV rGP with a 50% effective concentration (EC_50_) of <500 ng/mL.

The MAbs were further evaluated in a focus-forming assay (FFA) with BRBV for their ability to detect surface-expressed GP on infected cells. In cells infected with BRBV, only two of the nine tested MAbs (B02 and E02) were able to detect the GP protein on the surface of infected cells and produce distinct foci in this assay (Fig. 2E). The remaining seven MAbs were unable to detect GP expression in BRBV-infected Vero E6 cells. Of the two MAbs, E02 produced the most distinct and intense foci in the BRBV FFA (Fig. 2E). We determined the BRBV-neutralizing activities of these MAbs and found none of the antibodies neutralized the BRBV (data not shown). These data, combined with the ELISA data (Fig. 2D), demonstrate that we produced the first BRBV-specific monoclonal antibodies that can be used for diagnostic assays.

### A rapid, eGFP-based neutralization assay for BRBV.

**FIG 3:**
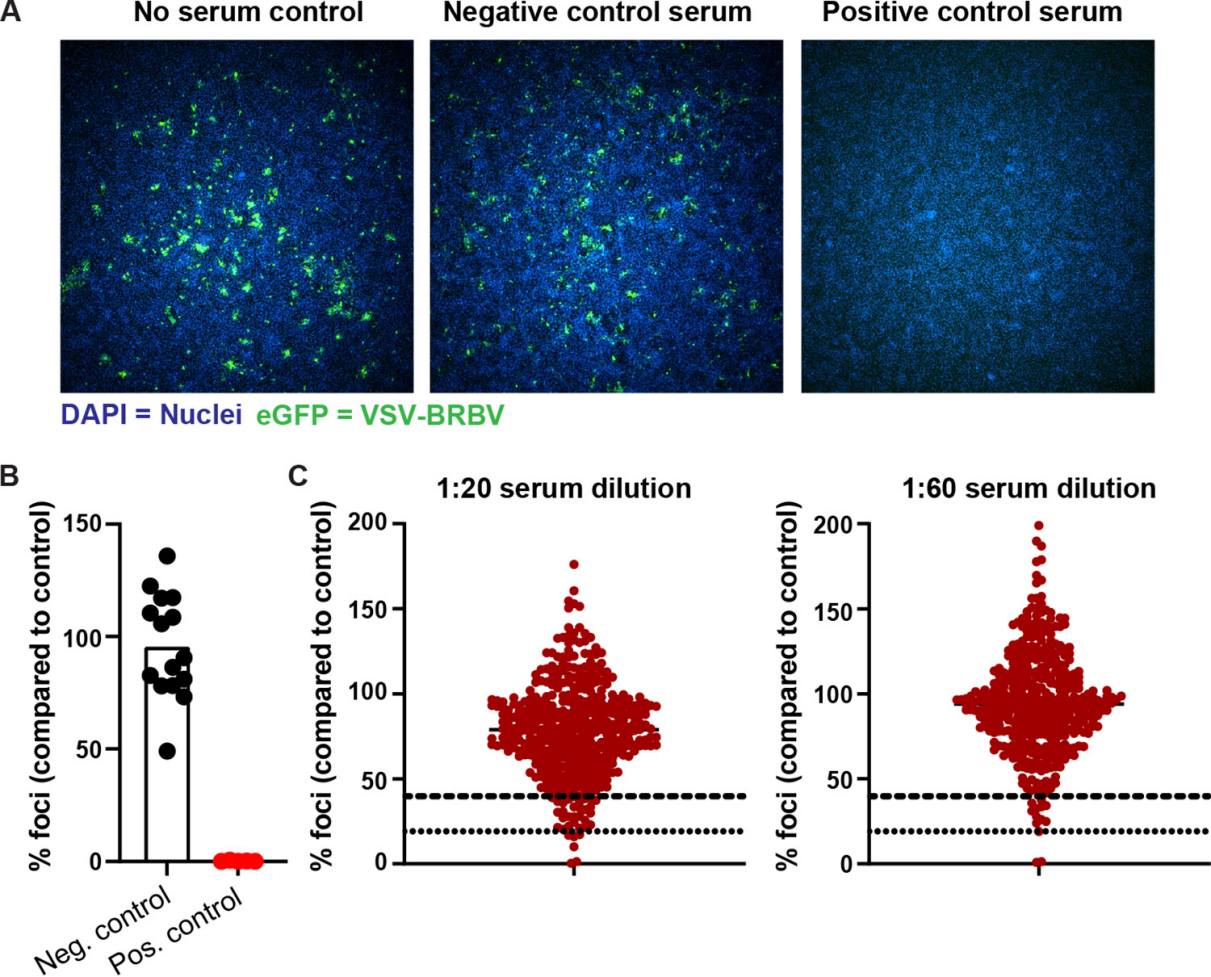
Development of a rapid eGFP-based BRBV neutralizing assay. (A) Effective neutralization of VSV-BRBV by BRBV-neutralizing antibodies. Monolayers of Vero cells incubated for 8 h with VSV-BRBV, expressing eGFP, in the presence of mouse serum-containing BRBV neutralizing antibodies (positive control), control mouse serum, or no serum were visualized with DAPI (blue). VSV-BRBV-infected cells were visualized by the expression of GFP from the VSV genome. Representative images of the no-serum (left), negative-serum (middle), and positive-serum (right) controls are shown at a magnification of ×4. (B) Normalized VSV-BRBV neutralization (percent foci compared to the no-serum control) of the positive- and negative-control sera. Each symbol represents an individual mouse serum sample (15 negative and 5 positive controls). (C) Normalized VSV-BRBV infection (percent foci compared to the no-serum control) of 440 human serum samples at a 1:20 (left) and 1:60 (right) dilution of the sera. Each symbol represents a serum sample. The dotted and dashed lines represent the cutoffs for 60% and 80% inhibition.

To facilitate throughput and reduce the costs associated with BSL3 work, we developed a rapid and high-throughput eGFP-based neutralization assay using a replication competent BRBV GP pseudotyped VSV that also expressed eGFP (VSV-BRBV). To demonstrate the utility of this virus, cells were inoculated with 200 FFU of VSV-BRBV with or without mouse sera containing neutralizing antibodies against BRBV. After 8 h without serum, eGFP expression is clearly visible in a subset of the cells (Fig. 3A, left). The addition of sera obtained from naive C57BL/6 mice did not inhibit the infection and replication of VSV-BRBV, and similar numbers of eGFP-positive cells were visible (Fig. 3A, middle). In contrast, the addition of serum collected 22 days after inoculation of C57BL/6 mice with 10^4^ infectious units of BRBV completely neutralized VSV-BRBV, and no eGFP-positive cells were detected (Fig. 3A, right). Analysis of the positive- and negative-control sera showed a significant difference (Student’s *t* test, *P < *0.0001) in neutralization of VSV-BRBV (Fig. 3B). Combined, these data show that VSV-BRBV can be used as a high-throughput alternative for live BRBV to detect BRBV-neutralizing antibodies.

A total of 440 human serum samples were tested in the rapid VSV-BRBV-based neutralization assay at two different dilutions of the sera (1:20 and 1:60). The inhibition of VSV-BRBV infection was normalized to the no-serum control wells included in each assay. The vast majority of the sera did not neutralize VSV-BRBV at either the 1:20 or 1:60 dilution (Fig. 3C). Interestingly, three human serum samples demonstrated >80% reduction in VSV-BRBV infection at both serum dilutions (Fig. 3C). In addition, 54 and 14 sera were identified as potentially positive (>60% but <80% inhibition) at the 1:20 and 1:60 dilutions of the sera, respectively (Fig. 3C). The 14 samples identified as potentially positive at the 1:60 dilution were also potentially positive at the 1:20 dilution and were selected for further testing in the BRBV focus reduction neutralization test (FRNT) together with 3 positive samples.

### Neutralization of BRBV by human serum samples.

**FIG 4:**
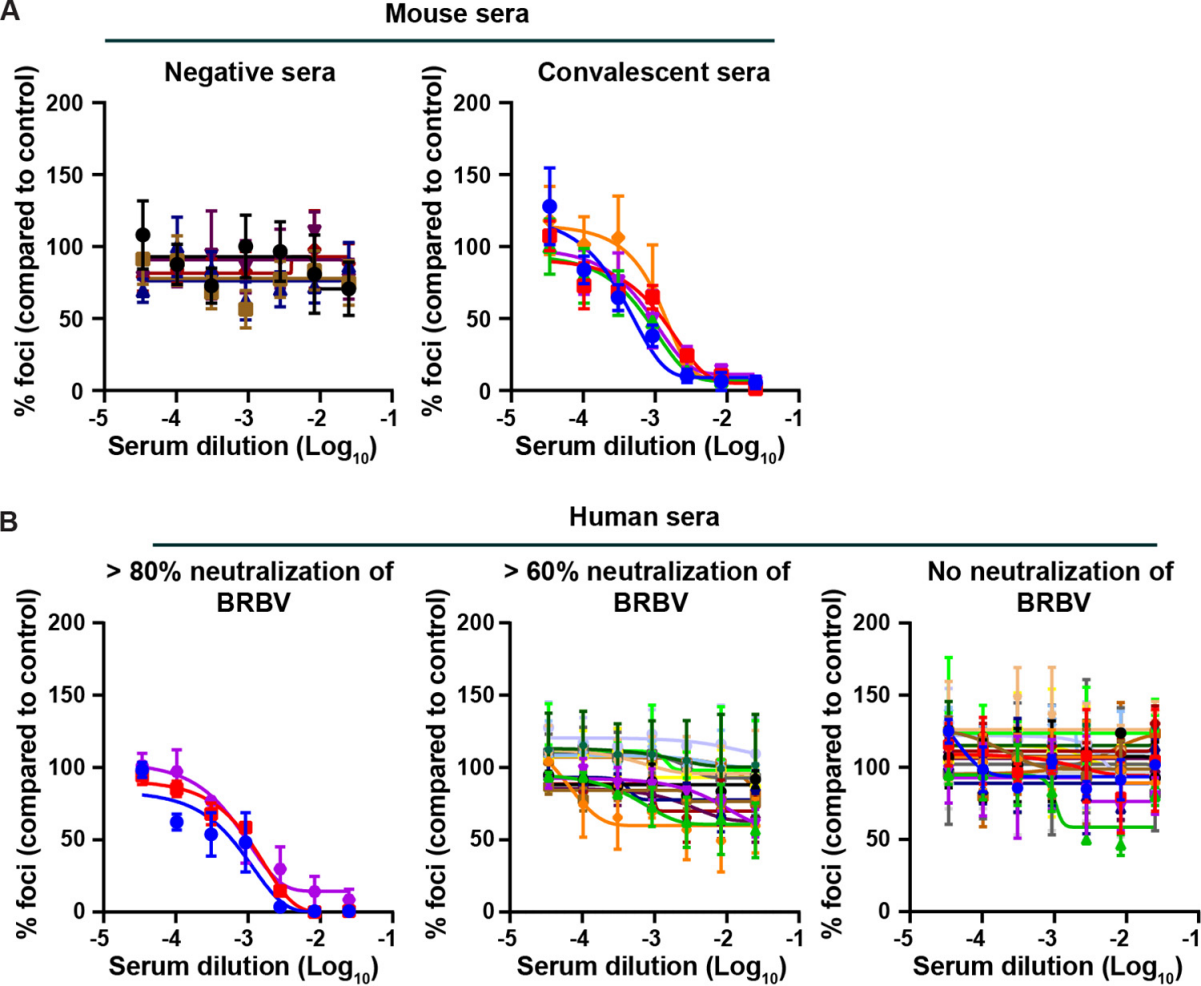
Detection of serum neutralizing antibodies against BRBV in human serum samples. A FRNT was developed for BRBV. Serially diluted heat-inactivated human or mouse serum (starting at a 1:40 dilution) was incubated for 1 h with 100 FFU of BRBV prior to being added to Vero cells. One hour later, the inoculum was removed, and the cells were overlaid with 1% methylcellulose and incubated for 24 h. Clusters of infected cells (foci) were visualized using the E02 MAb as described in Materials and Methods. The number of foci was normalized to the value for no-serum controls. (A) (Left) FRNT with serum from mock-infected control animals. No BRBV-neutralizing activity was detected in the negative-control sera. (Right) FRNT demonstrating virus-neutralizing activity in convalescent-phase serum collected from C57BL/6 mice 22 days after inoculation with 10^4^ PFU of BRBV. Neutralization of BRBV was detected in all five mouse serum samples. (B) FRNT on human serum samples with distinct neutralizing activities in the rapid assay. Human serum samples showing complete neutralization (0 to 20%) (left), partial neutralization (20 to 40%) (middle), or no neutralization (100%) (right) in the VSV-BRBV rapid assay were tested by FRNT. Three human sera were able to neutralize BRBV by FRNT.

To confirm our findings and demonstrate neutralization of live BRBV by antibodies in human serum, we developed a FRNT for BRBV using the BRBV GP-specific MAb (E02) described above. Sera collected from naive mice were unable to neutralize BRBV, and the number of foci was comparable to that in the no-serum controls (Fig. 4A, left). In contrast, convalescent-phase sera from mice infected with BRBV were able to potently neutralize live BRBV (Fig. 4A, right panel). The 50% inhibitory concentrations (IC_50_s) for these sera ranged between 1:500 and 1:1,350.

Next, the BRBV FRNT was used to test the 17 human serum samples that previously demonstrated complete or partial neutralization of VSV-BRBV (Fig. 3C). The three serum samples that strongly inhibited (>80% inhibition) VSV-BRBV also inhibited BRBV in the FRNT (Fig. 4B, left). The IC_50_s for these three serum samples were 1:2,631, 1:1,259, and 1:938. These three human serum samples were from males between 42 and 63 years old. The remaining 14 human serum samples that partially inhibited VSV-BRBV infection (>60% but <80% inhibition) did not neutralize BRBV in the FRNT (Fig. 4B, middle). As a control, we tested 19 human serum samples that did not neutralize VSV-BRBV. None of the 19 control sera were able to inhibit BRBV infection, and their IC_50_s were at the limit of detection (1:40) (Fig. 4B, right). Collectively, these data demonstrate that BRBV infections occur in the St. Louis community and that the seroprevalence of BRBV-neutralizing antibodies is ~0.7%.

## DISCUSSION

Here, we defined the first seroprevalence of BRBV serum neutralizing antibodies in human sera collected from the greater St. Louis metropolitan area. We developed two virus neutralization assays, a rapid VSV-BRBV eGFP-based neutralization assay and a BRBV focus reduction neutralization assay, to systematically evaluate the presence of BRBV-neutralizing antibodies in human serum. These assays identified 3 (0.7%) human sera that contained BRBV-specific neutralizing antibodies. These finding indicate that the incidence of BRBV infection in the St. Louis region in Missouri, USA, is higher than previously expected based simply on confirmed cases that have been reported.

BRBV is classified as a BSL3 pathogen, and work with this organism requires specialized laboratories, training, and equipment. As such, limited labs in the United States and around the world have the capacity to work with live BRBV. To facilitate the testing of BRBV GP-specific countermeasures and determine the seroprevalence of neutralizing antibodies in humans and wildlife, we developed a rapid eGFP-based neutralization assay using replication-competent VSV expressing both eGFP and BRBV GP. This virus can be used at a lower biosafety containment level (BSL2). Importantly, compared to previously described methods such as plaque reduction or neutralization testing (PRNT), both the eGFP-based rapid assay and the BRBV FRNT can be used to evaluate BRBV-neutralizing antibodies within 3 days. Thus, the turnaround time is greatly improved. Using sera from BRBV-immunized mice, we compared the EC_50_ between the VSV-BRBV rapid assay and the BRBV FRNT. We observed an approximately 5-fold-higher EC_50_ in the VSV-BRBV rapid assay than in the BRBV FRNT, suggesting that the VSV-BRBV rapid assay is more sensitive (data not shown) for the detection of BRBV-neutralizing antibodies.

One major limitation of studying emerging pathogens is the lack of reagents such as MAbs, recombinant proteins, reporter viruses, etc. This is the first report describing MAbs specific for GP of BRBV. Two of these MAbs were able to detect GP expression on cells infected with BRBV. Unfortunately, these antibodies did not neutralize BRBV, suggesting that they do not bind the receptor binding site of the virus (4).

BRBV and HRTV are classified as emerging infectious diseases that are transmitted via ticks in the United States. A previous study demonstrated a 0.9% seroprevalence of HRTV specific neutralizing antibodies in northwestern Missouri, US, where human cases and infected ticks have been identified (15). Our study demonstrates a 0.7% seroprevalence of BRBV-specific neutralizing antibodies in individuals in the St. Louis area. This is the first time that BRBV-neutralizing antibodies have been reported in individuals without a history of BRBV infection. These data also suggest that the true infection burden of BRBV in humans is higher than previously thought based on the five known cases of human BRBV infection. However, the seroprevalence in this cohort may underestimate the true infection rate and burden. First, our human serum cohort is from the greater St. Louis area and is likely composed primarily of urban populations that are less likely to be exposed to BRBV-infected ticks. Future seroprevalence studies with sera from individuals living in rural areas where BRBV infection in ticks have been reported are needed. Second, the presence of detectable levels of neutralizing antibodies is a relatively high threshold for determining prior infection with any virus, including BRBV. For example, there are many individuals previously infected with the influenza virus or SARS-CoV-2 who have no detectable serum neutralizing antibodies (16, 17). Moreover, the true infection burden of BRBV in the United States could be underestimated due to a lack of diagnostic testing, unknown virus geographical distribution, and unknown rate of asymptomatic infections. Combined with the widespread distribution of the lone star tick and the reported presence of BRBV-specific antibodies in North Carolina, we expect the true infection burden of BRBV to be much higher than what was previously expected based on the number of confirmed BRBV infections per year.

Three human sera showed the highest neutralization activity (>80% inhibition) in the VSV-BRBV eGFP-based assay. These same samples were confirmed as positive for BRBV-specific neutralizing antibody by FRNT. These findings suggest that the rapid assay and the BRBV FRNT are consistent in detecting the true seropositive samples and that our cutoff of 80% is rigorous. These two BRBV neutralization assays will expand our knowledge of BRBV seroprevalence in humans and in wild animals. Previous studies have shown that serum from white-tailed deer, raccoons, domestic dogs, horses, and eastern cottontails also contains BRBV-neutralizing antibodies (13, 14). The assays developed in this study will facilitate additional testing of animal species in areas where BRBV is endemic and where it is not and help identify the breadth of host species affected by this virus.

In conclusion, our study established two neutralization assays for BRBV and for the first time evaluated BRBV seroprevalence in a cohort of human sera. We found that 3 people in the St. Louis area were positive for BRBV-neutralizing antibodies.

## MATERIALS AND METHODS

### Virus and cell culture.

Vero E6 (ATCC) and Vero-CCL81 (ATCC) cells were maintained in Dulbecco’s modified Eagle medium (DMEM) containing 4.5 g/L glucose, l-glutamine, and sodium pyruvate (Corning) and supplemented with 10% fetal bovine serum (FBS; Biowest), 100 U/mL penicillin (Life Technologies), 100 μg/mL streptomycin (Life Technologies), and 2 mM l-glutamine (Corning). Expi293F cells (Thermo Fisher Scientific) used for transfection were maintained in Expi293 expression medium (Gibco).

VSV-BRBV was generated by replacing the endogenous G gene in a molecular cDNA of VSV, encoding eGFP, with a codon-optimized version of the BRBV GP gene (original strain, Kansas; GenBank no. AMN92169.1). Virus rescue was performed as described elsewhere (18). Briefly, BSRT7/5 cells were infected with vaccinia virus vTF7-3 and subsequently transfected with T7-driven helper plasmids encoding VSV N, P, L, and G and the VSV-BRBV cDNA. Cell supernatants were harvested after 72 h, centrifuged at 1,000 × *g* for 5 min, and passed through a 0.22-μm filter. Vero-CCL81 cells were then infected with rescue supernatants, and plaques in agarose plugs were isolated and passed to Vero-CCL81 cells for p1 stock generation. Working stocks were obtained by growing VSV-BRBV on Vero-CCL81 cells at a multiplicity of infection (MOI) of 0.01. RNA was extracted from virus stocks using the QIAamp viral RNA minikit. RNA was reverse-transcribed using the Qiagen OneStep RT-PCR kit with primers targeting VSV M-G (forward) and G-L (reverse) intergenic regions. Stock sequences were confirmed by Sanger sequencing. BRBV (strain BRBV-STL) (3) was grown on Vero E6 cells. Forty-eight hours after infection, the supernatant was collected and clarified by centrifugation, and aliquots were stored at −80°C. Virus stocks were sequenced by next-generation sequencing. A random hexamer was used to create cDNA for next-generation sequencing, and the library was prepared using the Illumina Nextera XT DNA library preparation kit. The amplified library was sequenced on a 2 × 150 MiSeq platform.

### VSV-BRBV characterization.

Vero-CCL81 cells were infected with eGFP-expressing VSV (referred to here as VSV) or VSV-BRBV at an MOI of 1. Cells were imaged 6 h postinfection (hpi). Plaque assays were performed by infecting Vero-CCL81 cells with the aforementioned viruses for 1 h at 37°C, after which the inoculum was removed, and cells were overlaid with an agarose-containing overlay. Plates were imaged 24 hpi using a biomolecular imager in the fluorescein isothiocyanate (FITC) channel.

### Recombinant proteins.

rGP (residues 20 to 485) was expressed using the baculovirus expression system or transiently expressed in mammalian cell lines. For expression with baculovirus expression system, the gene block of BRBV GP ectodomain followed by a trimerization sequence (GYIPEAPRDGQAYVRKDGEWVLLSTFL) from the bacteriophage T4 fibritin and a 6His tag at the extreme C terminus was cloned into a modified pOET1 (Mirus Bio) baculovirus transfer vector containing green fluorescent protein as an indicator (19). Transfection and amplification were carried out according to the *flash*BAC baculovirus expression system manual (Mirus Bio). The High Five (Gibco) cell culture supernatant was harvested after 72 hpi and concentrated before dialysis against the buffer 20 mM NaHCO_3_, 300 mM NaCl (pH 8.0). The BRBV GP ectodomain was captured by passaging the supernatant over Ni^2+^ affinity resin (GoldBio) and eluted with 500 mM imidazole (Sigma-Aldrich). The recovered proteins were further applied to a HiLoad 16/600 Superdex 200 column (GE Healthcare) in 20 mM HEPES, 150 mM NaCl at a pH of 7.5. For expression in mammalian cells, the gene fragment harboring the ectodomain, the trimerization domain as well as the 6His tag from the recombinant baculovirus vector was cloned into pFM1.2R vector (20) and transiently expressed in Expi293F cells. The cell culture supernatant was harvested after 5 days posttransfection and concentrated before being dialyzed against the buffer 20 mM Tris-Cl (pH 8.5), 150 mM NaCl. Then, the BRBV GP ectodomain protein was purified with the strategy described above, with a combination of Ni affinity chromatography and size exclusion chromatography.

### Animal experiments.

All animal experiments were preformed according to the Institutional Animal Care and Use Committee (IACUC) at Washington University in St. Louis. Eleven- to 12-week-old female C57BL/6 mice were obtained from The Jackson Laboratory. Mice (*n* *=* 3) were immunized intramuscularly with 500 ng of beta-propiolactone (BPL; Sigma-Aldrich)-inactivated and purified BRBV complemented with Addavax (InvivoGen) (1:1 [vol/vol]) to generate BRBV GP-specific MAbs. Four weeks later, the mice were immunized intramuscularly with 5 μg of baculovirus-derived recombinant GP of BRBV complemented with Addavax (1:1 [vol/vol]). Sera were collected 1 day prior to each immunization and stored at −20°C. To generate positive-control sera, containing BRBV-specific neutralizing antibodies, we infected C57BL/6 mice with 10^4^ infectious units of BRBV and collected sera at 22 days postinfection. Sera from five BRBV-infected mice were pooled to use as the positive control in the virus neutralization assays.

### Flow cytometry.

For B cell staining, spleen cells were collected 5 days after the final immunization and stained for 30 min on ice with CD16/CD32 (93; BioLegend; 1:100) and biotinylated mammalian expressed rGP (1:100) diluted in phosphate-buffered saline (PBS) supplemented with 2% FBS and 2 mM EDTA (P2). Next, the cells were washed twice with P2 and stained for 30 min on ice with anti-CD19–FITC (1D3; BioLegend; 1:100), anti-IgD–allophycocyanin (APC)-Cy7-A (11-26c.2a; BioLegend; 1:100), anti-Fas–phycoerythrin (PE)-A (Jo2; BD Biosciences; 1:200), anti-GL7–peridinin chlorophyll protein (PerCP)-Cy5.5-A (GL7; BioLegend; 1:50), anti-CD71–PE-Cy7-A (RI7217; BioLegend; 1:100), anti-CD138–BV421 (281-2; BioLegend; 1:100), anti-CD4–AF700 (GK1.5; BioLegend; 1:100), and Zombie Aqua (BioLegend; 1:200) diluted in brilliant stain buffer (BD Horizon). After 30 min, the cells were washed twice with P2, after which they were analyzed and single rGP-binding ASC (CD4^−^ CD19^+^ IgD^lo^ CD95^+^ GL7^−^ CD138^+^ BRBV GP^+^ live singlet lymphocytes) were sorted (*n* = 376) into four 96-well plates containing 2 μL of lysis buffer (Clontech) supplemented with 1 U/μL RNase inhibitor (New England BioLabs) with a FACSAria II instrument and immediately frozen on dry ice. Flow cytometry data were analyzed using FlowJo v.10.8.1.

### Monoclonal antibody production.

MAbs were generated as described previously (21). Briefly, VH, Vκ, and Vλ genes were amplified by reverse transcription-PCR (RT-PCR) and nested PCR from single-cell-sorted BRBV GP-specific ASC using mixtures of primer sets specific for IgG, IgM/A, Igκ, and Igλ. PCR products were then loaded on a 1% agarose gel (Lonza), purified, and sequenced by Sanger sequencing. Sequencing data were annotated using IMGT/V-QUEST v3.5.28 on the ImMunoGenetTics database (http://www.imgt.org/IMGT_vquest/) (22, 23). Clonally related cells were identified by similarity in variable heavy- and light-chain usage, CDR3 length, and amino acid composition. To generate recombinant MAbs, heavy-chain V-D-J and light-chain V-J fragments were PCR amplified from first-round PCR products with mouse variable-gene forward primers and joining J gene reverse primers having 59 extensions for cloning by Gibson assembly as previously described (24) and were cloned into pABVec6W Ab expression vectors (25) in frame with either the human IgG1, Igκ, or Igλ constant region. Expression plasmids were sequence (Sanger sequencing) verified and transfected at a 1:2 ratio of heavy to light chain into Expi293F cells using the ExpiFectamine 293 expression kit (Thermo Fisher Scientific). Culture supernatant was collected 7 days posttransfection, and MAbs secreted into the supernatant were purified with protein A agarose (Invitrogen) and then stored at 4°C until further use.

### ELISA.

Ninety-six-well microtiter plates (Thermo Fisher Scientific) were coated overnight at 4°C with 1 μg/mL of baculovirus-expressed rGP in PBS. After the wells had been washed three times with 280 μL PBS-T (PBS supplemented with 0.05% Tween 20), they were blocked with 280 μL of PBS supplemented with 10% FBS and 0.05% Tween 20 (blocking buffer) for 1.5 h at room temperature. Blocking buffer was removed, and 3-fold serial dilutions starting from 1:30 for mouse polyclonal sera or 30 μg/mL for MAbs were added and incubated for 1 h at room temperature. An influenza A virus-specific MAb (1C11) specific for the hemagglutinin of A/Puerto Rico/8/1934 was used as a negative control. Plates were washed three times with PBS-T. MAbs were detected using goat-anti-human IgG conjugated to horseradish peroxidase (HRP) (Jackson ImmunoResearch; catalog no. 109-035-088), while mouse sera were detected using goat-anti-mouse IgG1-HRP (Southern Biotech; catalog no. 1070-05) diluted 1:1,000 in blocking buffer for 1 h at room temperature. After three washes with PBS-T followed by three washes with PBS, the assay was developed with 100 μL of substrate solution (phosphate-citrate buffer with 0.1% H_2_O_2_ and 0.4 mg/mL *o*-phenylenediamine dihydrochloride [OPD]; Sigma-Aldrich]) was added to all wells and incubated for 5 min before the reaction was stopped with 1 M HCl (100 μL). Optical density measurements were taken at 490 nm using a microtiter plate reader (BioTek).

### FFA.

Monolayers of Vero E6 cells, seeded in 96-well tissue culture treated plates, were washed once with PBS and incubated with VSV-BRBV or BRBV diluted in serum-free DMEM (Corning). After 1 h at 37°C, the inoculum was aspirated, the cells were washed with PBS, and 100 μL of 1% methylcellulose (Sigma) in 1× minimum essential medium (MEM; Corning) supplemented with 2% FBS was added to each well. The cells were incubated for 24 h at 37°C and 5% CO_2_ before the monolayer was fixed with 100 μL of 5% formalin (Fisher Chemicals) for 1 h at room temperature. Cells were washed with PBS and subsequently incubated with the BRBV GP-specific MAb E02 in PBS-T plus 1% bovine serum albumin (BSA; Sigma). After 1 h of incubation at RT, the cells were washed three times with PBS-T and incubated with HRP-conjugated goat anti-human IgG (Sigma) diluted in PBS-T plus 1% BSA for 1 h at room temperature. Finally, cells were washed three times with PBS-T, and VSV-BRBV- or BRBV-infected cells were visualized using 3,3′,5,5′-tetramethylbenzidine (TMB) substrate (Vector Laboratories) and quantitated on an ImmunoSpot analyzer (Cellular Technologies).

### Human serum cohort.

We screened 440 adult serum samples that were part of a set of residual samples sent to Barnes-Jewish Hospital, Missouri, for physician-ordered vitamin D testing between 27 April 2020 and 12 May 2020 (26). This study was approved by the Human Research Protection Office at Washington University in St. Louis (approval no. 202004199).

### Rapid eGFP-based VSV-BRBV GP virus neutralization assay.

Vero E6 cells were cultured overnight in tissue culture treated black 96-well plates (Corning). Human sera were heat inactivated at 56°C for 30 min and serially diluted 1:10 and 1:30 in serum-free DMEM (DMEM-SF). The next day, 200 FFU of VSV-BRBV diluted in an equal volume of DMEM-SF was added to the diluted human serum samples (final serum dilutions, 1:20 and 1:60) and incubated for 1 h at room temperature. Next, the antibody virus complexes were added to the Vero E6 cells, which were washed once with PBS and incubated for 8 h at 37°C. Each serum sample and dilution was tested in duplicate, and each assay included a positive-control mouse serum sample and no-serum controls (DMEM only). After 8 h, the cells were fixed with an equal volume of 10% formalin containing 10 μg/mL Hoechst (Sigma), which was added to each well for 1 h at room temperature. Subsequently, cells were washed once with PBS, and images were acquired with the InCell 2000 analyzer (GE Healthcare). This automated microscope contains both FITC and DAPI (4′,6-diamidino-2-phenylindole) channels to visualize infected cells (i.e., eGFP-positive cells) and nuclei, respectively. Each well is divided into 4 fields that are imaged with a 4× lens objective. Subsequently, cumulative eGFP-positive cells and nuclei of the 4 fields were counted. Images were analyzed using the Multi-Target Analysis module of the InCell Analyzer 1000 Workstation software (GE Healthcare). eGFP reduction neutralization activity of the serum samples was calculated by dividing the number of eGFP-positive cells for each serum sample by the average number of eGFP-positive cells in the no-serum control wells.

### BRBV neutralization assay.

A FRNT was developed for BRBV. Vero E6 cells were seeded overnight in tissue-cultured treated 96-well plates (Corning). Heat-inactivated human or mouse sera were diluted 1:20 in DMEM-SF and subsequently serially diluted 3-fold to a final dilution of 1:14,580. Next, an equal volume of DMEM-SF containing 200 FFU of BRBV was added to each serum dilution and incubated for 1 h at room temperature. The addition of the virus resulted in a final serum dilution of 1:40 to 1:29,160. Next, the Vero E6 cells were washed once with PBS, and the antibody-virus complexes were transferred to cells and incubated for 1 h at 37°C. Each serum sample and dilution was tested in duplicate, and each assay included positive (pooled convalescent-phase sera from BRBV-infected mice), negative serum, and no-serum controls. After 1 h at 37°C, the cells were washed once with PBS, and 100 μL of MEM containing 2% FBS and 1% methylcellulose was added to each well. After 24 h at 37°C, the cells were fixed by adding 100 μL of 10% Formalin on top of the overlay (final concentration, 5% formalin) for 1 h at room temperature. Subsequently, cells were washed with PBS and the assay was developed as described above for the focus-forming assay. The percent inhibition of BRBV infection was calculated by dividing the number of foci in the test sample by the average number of foci in the no-serum control wells.

### Statistical analysis.

Data were analyzed by using GraphPad Prism version 9.3.1 software. EC_50_s and IC_50_s were calculated by using log(inhibitor) versus response in variable slope (four parameters), fixing both bottom and top constraints at 0 and 100, respectively.
